# Dicrocoelium Egg Identified in an Ancient Cemetery in Kiasar Archeological Site, Northern Iran, Dated Back 247 BC–224 AD

**Published:** 2017-06

**Authors:** Negar BIZHANI, Abdol Motalleb SHARIFI, Mohmmad Bagher ROKNI, Jean DUPOUY CAMET, Mostafa REZAEIAN, Mohammad FALLAH KIAPI, Niloofar PAKNEZHAD, Faezeh NAJAFI, Gholamreza MOWLAVI

**Affiliations:** 1. Dept. of Parasitology and Mycology, School of Public Health, Tehran University of Medical Sciences, Tehran, Iran; 2. Center of Research, Office of Cultural Heritage, Handicrafts and Tourism Organization of Mazandaran, Sari, Iran; 3. Center for Research of Endemic Parasites of Iran (CREPI), Tehran University of Medical Sciences, Tehran, Iran; 4. ACMSFI, Hôpital Cochin, 27 Faubourg St Jacques, 75014 Paris, France; 5. Research Organization of Sarishenasi, Sari, Iran

**Keywords:** Parasite, Paleoparasitology, *Dicrocoelium dendriticum*, Iran

## Abstract

**Background:**

Along with the newly emergence of paleoparasitology research in Iran, findings of parasites from Northern part of the county have not been reported so far. In this study tracking for the lancet liver fluke dates back 250 BC is addressed.

**Methods:**

Samples were taken from grave crypts of the soil layers attached to the pelvic bones from above-mentioned site in 2015. The laboratory examinations were conducted in the Dept. of Medial Parasitology and Mycology, School of Public Health, Tehran University of Medical Sciences, Tehran, Iran. Current rehydration technique using TSP 0.5% was utilized for examining the samples.

**Results:**

Out of 10 burial soil samples examined, one individual was seen parasitized with a *Dicrocoelium* egg. The burial belonged to an adolescent male 20–22 yr old. The egg was in brown color and the length/width parameters of 36×22/5 μm. Parthian coins found in nearby the burials in Kiasar Cemetery, declared the time of the skeleton about 247 BC – 224 AD confidently.

**Conclusion:**

Although the possibility of transit infection with *D. dendriticum* is high, yet the environmental and geographical conditions in that time are in favor of a normal human transmission in northern Iran.

## Introduction

Paleoparasitological research describes different aspects of parasitic infections such as host parasite relationships, the origin of parasites and the patterns of transmission to humans over the time. *Dicrocoelium* genus Dujardin, 1845 and predominantly *D. dendriticum* is the causative agent of dicrocoeliasis, a zoonotic liver fluke infection of a worldwide distribution (1). Different herbivores, frequently sheep, are its natural definitive host and terrestrial snails and ants play as the first and the second biological intermediate hosts in *Dicrocoelium* life cycle respectively (1). Humans may, unfrequently, acquire the infection through the ingestion of raw vegetables containing infective metacercariae anchored in ants (2). Transmission in animals occurs while grazing.

Eggs finding through coprological studies in ruminants, indicates a real *Dicrocoelium* infection, while human positive cases cannot be confirmed by one time stool examination, due to spurious infection, which can be happened following the consumption of ruminant infected livers (3). Paleoparasitological evidence reveals the antiquity of *Dicrocoelium* spp. since 550,000 years BP in Europe (4). Review of the literature shows the records of *Dicrocoelium* spp. in several archeological sites from different regions in the ancient world (5).

The study of parasites of ancient times is a new line of research in Iran attested by the finding of several helminth eggs as well as *D. dendriticum* egg in a cemetery of the Bronze Age in southwestern Iran (6).

Similarly, the present paper, reports the finding of *D. dendriticum* egg in Kiasar archeological site in Caspian Sea littoral in northern Iran dates back 250 BC.

## Materials and Methods

**Fig. 1:**
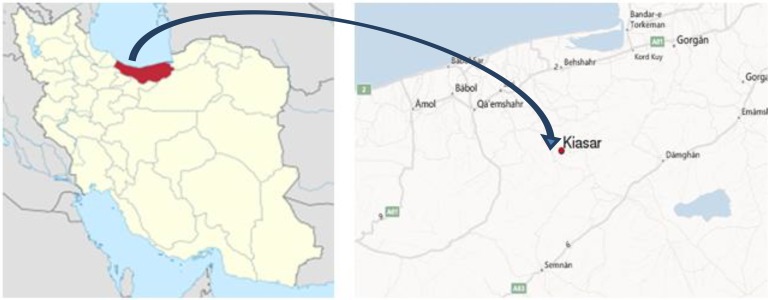
Kiasar Archeological site, Northern Iran

Kiasar archeological site dated back to the Parthian dynasty (250 BC), is located in Mazanderan Province (N: 36 14′ 317″ E: 053 35′ 149») on the Caspian Sea littoral of Iran (Fig. 1).

Samples were mostly collected in grave crypts in 2015 from the soil layers attached to the pelvic bones while all laboratory examinations were performed in the School of Public Health, Tehran University of Medical Sciences, Tehran, Iran.

Current rehydration technique using TSP 0.5% was applied for the samples (7). Microscopic slides were mounted using glycerin gel and the found case photographed by a Digital Microscope Camera Olympus Dp12 (Hamburg, Germany). Identification was carried out consequent to morphometric measurement compared with referral descriptions in textbook of helminthology (8).

## Results

**Fig. 2:**
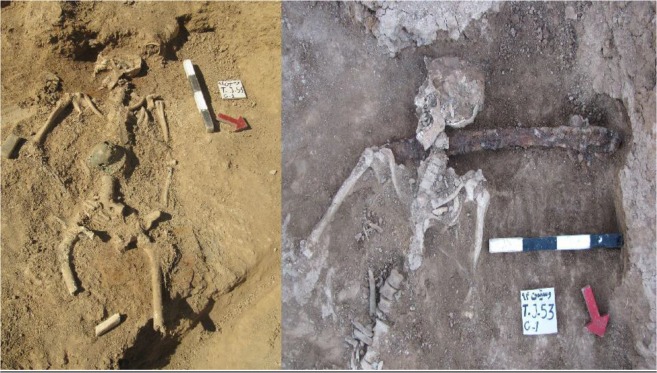
The grave and the parasitized Skeleton with *Dicrocoelium* egg from different views in Kiasar site. The sword beneath the neck illustrates the appearance of an army member

The burial soils obtained from the graves were analyzed within which a sample of a 20–22 yr old male was seen parasitized by *Dicrocoelium* egg (Fig. 2).

**Fig. 3:**
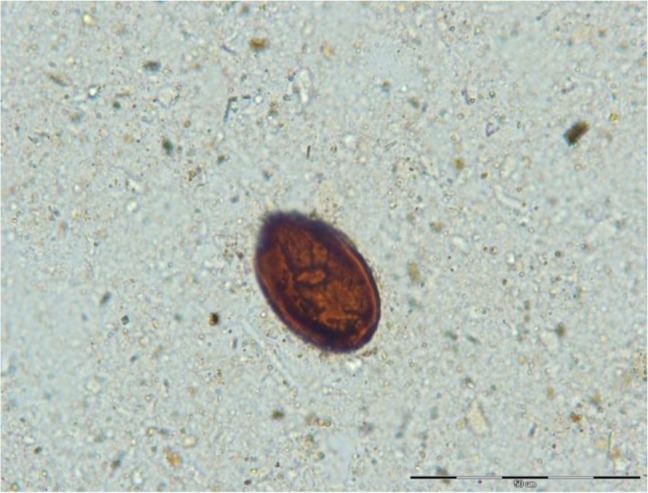
*Dicrocoelium* egg detected under the microscope, scale bar (50 μm)

The morphological characteristics (brown color, thick shell and a 36×22.5 μm in size) did match to a *Dicrocoelium* egg most probably, *D. dendriticum* (Fig. 3). From the perspective of archeologists, the young soldier must have been killed in a battle since a sword and other personal war belongings were observed around the sampled skeleton (Fig. 2). Parthian coins found in nearby the burials in Kiasar Cemetery, declared the time of the soldier about 247 BC – 224 AD confidently.

## Discussion

Dicrocoeliasis caused by trematode helminths from the genus *Dicrocoelium*, so called lancet liver fluke.

The parasite circulates amongst variety of herbivores and occasionally in humans with terrestrial snails and ants playing as biological intermediate hosts. Ingestion of the infected ants harboring metacercarial stage is fundamental in infection transmission for the both humans and animals (9).

Confirmation of a real dicrocoeliasis in ancient humans is a controversial issue, since the consumption of herbivore’s infected liver carrying a great number of eggs could be manifested as spurious infection (10). This condition in *Dicrocoelium* egg passers, is a concern for clinical laboratories nowadays.

Based on paleoparasitological records, the antiquity of dicrocoeliasis on the earth can be backed to 550,000 years ago (4) in an isolated coprolite from the Middle Pleistocene context.

Since the Neolithic period when domestication and agriculture begun, the chance of zoonotic infection transmission to humans gradually increased, owing to closeness of animal to human surroundings on that time (11). The existence of *Dicrocoelium* sp and *Fasciola hepatica* eggs in European wetlands in Switzerland and France have been documented in this era (12). In the old world, ancient eggs of *Dicrocoelium* sp. have been found in the African continent, from coprolites in K2 archaeological site, in South Africa (13). In the first paleoparasitological findings in Newfoundland, Canada, *Dicrocoelium* eggs among those of other helminths, have been detected in the remains of an old fashioned toilet belonged to 17^th^ century (14). In this paper the found eggs could not be attributed to human origin due to possible contamination with the animal feces of the nearby stable.

In our present case, the both spurious and real infection can be justified. Nevertheless, the occurrence of human dicrocoeliasis in northern Iran on that time can be assumed prevalent based on expected environmental and geographical conditions. Nowadays northern provinces of Iran, Mazandaran and Gilan are known endemic, for the lancet liver fluke infection (15, 16).

Further studies on this ancient site with the use of modern techniques could lead us to a comprehensive interpretation about the health level and the status of zoonotic infections in Parthian era (250 BC).

## Ethical considerations

Ethical issues (Including plagiarism, informed consent, misconduct, data fabrication and/or falsification, double publication and/or submission, redundancy, etc.) have been completely observed by the authors.
